# An Evaluation of the Binding Strength of Okra Gum and the Drug Release Characteristics of Tablets Prepared from It

**DOI:** 10.3390/pharmaceutics9020020

**Published:** 2017-06-02

**Authors:** Amjad Hussain, Farah Qureshi, Nasir Abbas, Muhammad Sohail Arshad, Ejaz Ali

**Affiliations:** 1 University College of Pharmacy, University of the Punjab, Lahore 54000, Pakistan; farah.qureshi@lmdc.edu.pk (F.Q.); nasirabbas77@gmail.com (N.A.); ejazali502@hotmail.com (E.A.); 2 Faculty of Pharmacy, Bahauddin Zakariya University, Multan 60800, Pakistan; sohailarshad@bzu.edu.pk

**Keywords:** okra gum, binding, strength, drug release

## Abstract

The aim of this study is to evaluate the adhesion ability of okra gum, which is gaining popularity as a tablet binder. For this purpose, gum was extracted from okra pods, and the binding strength of different concentrations (1%, 3%, and 5%) was determined quantitatively. Additionally, naproxen sodium tablets were prepared by using okra gum as a binder and were evaluated for their properties including hardness, friability, disintegration time, and dissolution rate. The binding strength values were compared with that of pre-gelatinized starch, a commonly used tablet binder. The results from universal testing machine indicate that the binding strengths of all dispersions of okra increase as the concentration increases from 1% to 5% and ranges from 2.5 to 4.5 N, which are almost twice a high as those of pre-gelatinized starch. The tablets prepared with okra gum have shown good mechanical strength with hardness values of 7–8.5 kg/cm^2^ and a friability <1%, comparable to tablets prepared with starch. The disintegration time was longer (7.50 min with okra gum and 5.05 min with starch paste), and the drug release from these tablets was slower than the formulations with starch. The higher binding ability of okra gum probably linked with its chemical composition as it mainly contains galactose, rhamnose, and galacturonic acid. This study concludes that okra gum is a better binder than pre-gelatinized starch, it might be explored in future for introduction as a cost-effective binder in the pharmaceutical industry.

## 1. Introduction

Binders are a major class of excipients used to hold the active pharmaceutical ingredient (API) and inactive excipients together in a cohesive mass [1]. These are also called granulating agents and promote size enlargement to help build granules of desired sizes, thereby enhancing the free flowing qualities of the powder for a solid dosage form [2]. The presence of a binder also enables intergranular binding and ensures that the tablet remains intact after its compression by improving its crushing strength and reducing friability [3]. The nature and concentration of a binder influences the characteristics of a compressed tablet. There are a wide variety of substances that are used as binders in tablet formulations. Generally, they can be categorized into three groups: (i) sugars, such as sucrose, glucose, sorbitol; (ii) natural gums and polymers, which include pre-gelatinized starch, starch, gum acacia, gelatin, and sodium alginate; and (iii) synthetic polymers, which include PVP, PEG, and all the semisynthetic derivatives of cellulose (HPMC, HPC, CMC, EC, and polymethacrylates) [4].

The use of natural binders is more advantageous than the use of synthetic ones because of the natural binders’ easy availability, low cost, biocompatible, biodegradable, and environmentally friendly nature [5]. Natural gums commonly used as tablet binders are Khaya gum [6], Leucaena leucocephala seed gum, Anacardium occidentale gum, Gellan gum, and gum acacia [7]. Okra gum is one of such gums and has gained popularity over the last few decades in pharmaceuticals. In solid dosage forms, it has been shown to be a promising excipient for the modification of drug release from tablets of different APIs [8]. Furthermore, its properties as a tablet binder [9,10] and film coating [11] have been evaluated in many studies.

In liquid dosage forms, okra gum has been used as an emulsifying agent/stabilizer in emulsions [8,12] and as a suspending agent in pediatric suspensions [13]. Okra gum has also been used in nasal drug delivery systems as a mucoadhesive and for its gel forming properties [14]. Earlier studies have compared the binding ability of okra gum with that of PVP, cornstarch [15], and gelatin [10] and have shown that okra gum is a better binder than these routinely used binders. However, there is no study signifying the binding strength of okra gum in quantitative terms.

In this study, the binding strength of okra gum was determined by two different methods, using the principle whereby the two surfaces, which were previously attached by applying adhesive material between them, are detached by applying the load, . The binding ability of okra gum was also demonstrated in terms of the characteristics of granules and tablets prepared by wet granulation using naproxen sodium as a model drug and different concentrations of okra gum as a binder. The characteristics that were studied include the bulk properties of granules as well as the tablet’s hardness, friability, disintegration time, and drug release patterns. The results were compared with tablets prepared using a classical binder, i.e., pre-gelatinized starch, as it is a commonly used binder in the pharmaceutical industry. 

## 2. Materials and Methods

Fresh pods of *Abelmoschus esculentus* (okra) were purchased from the local market (Lahore, Pakistan). Naproxen sodium was obtained as a gift sample from Pacific pharmaceuticals Pvt (Ltd.), Lahore, Pakistan. The excipients including starch (boiled with water to obtain pre-gelatinized starch), lactose, magnesium stearate, and talc were obtained from local suppliers (Lahore, Pakistan), and these were of pharmaceutical grade. Starch used in this study was cornstarch, which generally contains amylose and amylopectin (major constituents). The organic solvents including chloroform, acetone, ethyl acetate, and hexane were purchased from Merck, Germany.

### 2.1. Preparation of the Aqueous Extract of Okra

Okra gum was extracted from the fresh pods of the *Abelmoschus esculentus,* by adopting the extraction method as already described in the literature [16]. For this purpose, okra pods (~0.25 kg) were washed, dried at room temperature (~25 °C), and sliced horizontally into small ~1 inch pieces after the calyces were removed. These slices (weighing 0.10 kg) were taken in a pan, ~1.5 L of distilled water was added, and they were then heated at ~60 ° C for 4 h with intermittent stirring. The mixture was filtered through a muslin cloth, and the aqueous extract was cooled at 4–6 °C in a refrigerator.

### 2.2. Isolation of Okra Gum

From the aqueous extract, the gum okra was precipitated by adding three ~250 mL portions of acetone and filtering the precipitates each time using filter paper. The precipitate was dried in an oven at ~40 °C until a constant weight was achieved. The dried gum was crushed in a pestle and mortar, passed through a mesh of sieve number 20, and stored in an air-tight glass container at room temperature until further use.

### 2.3. The Determination of Binding/Adhesive Strength

For the measurement of binding/adhesive strengths, three different concentrations (1%, 3%, and 5%) of both binders (pre-gelatinized starch and okra) were prepared by dispersing the accurately weighed quantities of each binder in distilled water. The binding strength was measured by two different methods; both have almost the same principle whereby the two surfaces, attached by smearing a thin layer of adhesive material between them, are detached by applying the load. The magnitude of load represents the binding strength of the adhesive material applied. The first method uses a modified balance apparatus named as a double pulley assembly, while the second applies a well-known universal materials testing machine.

#### 2.3.1. Testing Using the Double Pulley Assembly/Modified Balance Apparatus

**Figure 1:**
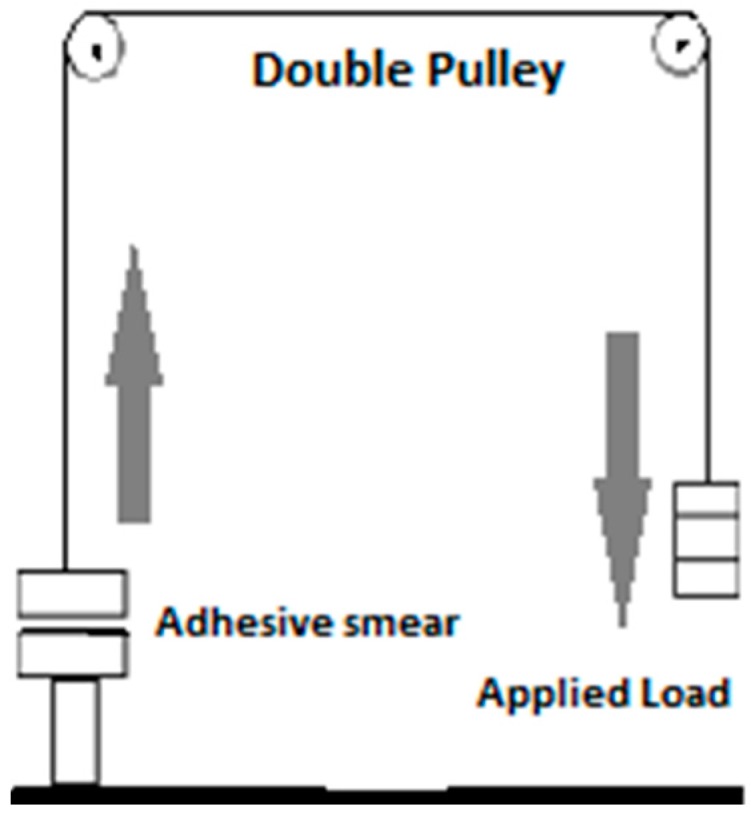
Illustration of determination of binding strength by double pulley apparatus.

The double pulley apparatus (locally assembled) consists of two freely moving pulleys supported by a brass frame on both sides (Figure 1). At the base of the left pulley stands a glass slide attached on a brass support. A stretch-less string was attached to a hanging glass slide at one end and with a weight carrier hook on the other end. This string passes over both pulleys so that the end, which has a hanging glass slide, comes on the lower glass slide, and the string is balanced with the weight on the right side.

For measuring the binding strength, a thin smear of constant surface area, i.e., 2 cm^2^ was applied on the lower slide. The upper slide was joined to the lower slide so that the film was sandwiched between both slides. The load was applied on the weight hook until both slides were detached from each other. This total load represents the binding strength of the adhesive smear. The values were recorded in triplicate (*n* = 3), and the mean was calculated. An almost similar apparatus and method has already been used for measuring mucoadhesive strength in literature [17,18,19].

#### 2.3.2. Universal Materials Testing Apparatus

The binding strength of both binders was also determined in a universal material testing machine (Lloyd Lf Plus series, Lloyd Instruments Ltd., Largo, FL, USA). The lower jaw of the machine was affixed with a glass slide on its top to make a fixed base, while another glass slide was hung from the upper jaw using a strand of thread. An area of 2.5 cm^2^ was marked on the fixed lower slide, a thin smear of the sample was made in the marked area, and the upper jaw was lowered down to such a distance that the upper glass slide attaches with the lower one. 

Tear and peel test from nexygen MT-materials testing software was applied and the jaws moved at the speed of 250 mm/s. The force required to separate both the slides was the binding strength of the applied material. Each measurement was recorded in triplicate, and the average value was reported.

### 2.4. Preparation of Tablets

The tablets of naproxen sodium containing either okra gum or pre-gelatinized starch as a binder in the similar concentrations (1%, 3%, and 5%) were prepared by wet granulation. A set of twelve formulations was prepared; six were made using okra gum as a binder. The first three formulations (F1–F3) were without any disintegrant and the next three (F3–F6) had 7% starch as disintegrant (Table 1). The other six formulations were prepared using pre-gelatinized starch as a binder; of these, three formulations F7–F9 were prepared without any disintegrant, while the remaining three formulations F10–F12 had starch as a disintegrant (Table 1).

The effect of the concentration of both binders in the presence or absence of disintegrant on the properties of tablets including hardness, disintegration time, and the release of drug was studied.

### 2.5. Bulk Properties of Granules

The bulk and tapped density of granules were measured in order to assess their flow and compressibility properties. From the value of bulk and tapped density, the Hausner ratio and the compressibility index or Carr′s index was calculated by applying Equations (1) and (2), respectively.

(1) $$\mathrm{Hausner}\;\mathrm{ratio}=\frac{\mathrm{Tapped}\;\mathrm{denstiy}}{\mathrm{Bulk}\;\mathrm{density}\;}.$$

(2) $$\mathrm{Compressibility}\;\mathrm{index}=\;\frac{\mathrm{Tapped}\;\mathrm{density}-\mathrm{bulk}\;\mathrm{density}}{\mathrm{Tapped}\;\mathrm{density}\;}\times 100.$$

### 2.6. Testing of Prepared Tablets

The prepared tablet formulations were evaluated for their physical properties including hardness, friability, and disintegration time according to official methods as described in United States Pharmacopoeia (USP).

### 2.7. Drug Release Studies

In order to study the release pattern of naproxen sodium from the tablet formulations prepared with different concentrations of both binders, in vitro dissolution studies were performed using USP type II apparatus (Curio, Pakistan). Phosphate buffer (900 mL) at pH 7.4 was used as dissolution medium. The temperature of the dissolution medium was maintained at 37 ± 0.5 °C, and the paddle rotating speed was set at 50 rpm. Aliquots of 5 mL were withdrawn from the dissolution vessel at predetermined time intervals. These were diluted adequately and analyzed for drug content by a UV spectrometer (2550 Schimadzou, Kyoto, Japan) at 331 nm. (λ_max_ of the naproxen sodium determined in this study. It was also observed that the okra gum solution does not show any significant absorbance at this wavelength, so there are least chances of interfering with assay results). A similar method has been used previously [20].

## 3. Results

The extracted okra gum (yield ~2%) was a dark brown colored powder with strong unpleasant odor. Its dispersion in water was a brownish; translucent that is sticky in nature. The values of binding strength of this dispersion as measured by two different methods have been described below.

### 3.1. Binding Strength of Okra Gum and Pre-Gelatinized Starch

The values of binding strengths of dispersions of okra gum and pre-gelatinized starch in three different concentrations, i.e., 1%, 3%, and 5% in water have been summarized in Table 2. The results show that the values of binding strength obtained from the two measurement methods were not consistent with each other, representing the variation between the measuring techniques. However, a similar trend of increasing binding strength as binder concentration increases from 1% to 5% for both binders was observed with both measurement techniques. Additionally, it is interesting to note that the binding strength of okra gum in similar concentrations is comparable or even better than that of pre-gelatinized starch, a routinely used tablet binder.

In the next part of this study, the binding ability of okra gum is demonstrated using the same three concentrations in designing the tablet formulations (which in turn were characterized in terms of hardness, friability, disintegration time, and release of drug). These formulations were then compared with those prepared using starch as a binder.

### 3.2. Bulk Properties of the Granules

The bulk density values of granule formulations prepared with okra gum (F1–F6) were in the range of 0.42–0.43 g/cm^2^. On the other hand, for formulations of starch (F7–F12), these were between 0.45 and 0.48 g/cm^2^. While the tapped density values of respective granules were 0.46–0.48 and 0.52–0.56 g/cm^2^ (Table 3). The Hausner ratio calculated from these values lies in the range of 1.10–1.18, indicating an excellent flow behavior of the granules. Similarly, the compressibility index values of the majority of the formulations are between 1 and 10, indicating good compression behavior [21].

### 3.3. Characteristics of Prepared Tablets

The tablets prepared with okra gum as a binder with different concentrations have shown good characteristics in terms of hardness, with 6.5–8.75 Kg/cm^2^, a friability of <1%, and a disintegration time between 5 and 15 min (Table 4). These characteristics are within the official limit set for the each. It is interesting to note that the values of friability and hardness of tablet formulations do not change as the concentration of both binders varies, while the disintegration time increases as the concentration of both binders increases, with values of ~8 min and 6 min with 1% okra and starch, respectively, which is ~15 min with 5% of both binders (Table 4). Furthermore, the addition of starch powder as a disintegrant in F4–F6 and F10–F12 caused a little reduction in the value of disintegration time. 

### 3.4. Dissolution Studies

**Figure 2:**
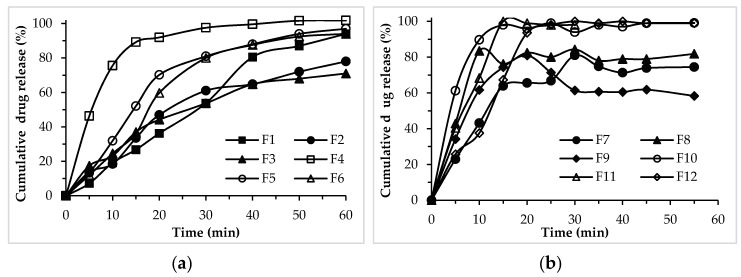
Release profile of naproxen sodium from the tablets prepared using different concentrations of okra gum F1 to F6 (**a**) and pre-gelatinized starch F7 to F12 (**b**) as a binder. The profiles with an open marker represents the formulation without disintegrant, while the filled marker profiles show the formulation containing disintegrant.

The dissolution profiles of naproxen sodium in phosphate buffer (pH 7.4) from the formulations prepared using okra gum as a binder in three different concentrations are shown in Figure 2. The formulations without disintegrant (F1–F3) show a 25–35% of drug release in the first 15 min, which reaches 50–60% in 30 min and 70–90% in 60 min. It is also evident from the results that drug release at each time point decreases as the concentration of the binder in the formulation increasing.

The formulations containing disintegrant (F4–F6) show a relatively faster release of drug, i.e., 40–80% in the first 15 min and >80% release within 30 min. These results show that, in the presence of a disintegrant, okra gum behaves as a binder with drug-releasing properties similar to those of pre-gelatinized starch (Figure 2b). However, in the absence of a disintegrant, the release profile of the drug is sustained from the formulation. 

## 4. Discussion

The binding strength results from both measurement methods show that okra gum is a better binder for naproxen sodium tablets than pre-gelatinized starch, the commonly used tablet binder. The double-pulley method yielded lower values of binding strengths for all concentrations of both binders than the universal testing method. This probably represents the inability of the former to correctly measure the adhesiveness. This argument is based on the fact that weight increments in this apparatus added to detach the joined surfaces were not precisely controlled as compared with the universal testing machine. This also resulted in higher standard deviation in these values. Therefore, the binding strength values, based on the universal testing machine, were considered relatively more reliable. The results show that these values for okra gum are almost twice as high as those for starch. The binding ability of okra is probably better than starch because of the difference in molecular structure of both polymers. In okra gum, the major constituents galactose, rhamnose, and galacturonic acid have more adhesion potential based on their ester crosslinking nature [22], while starch consists mainly of glucose monomers with alkyl linkages. These findings may present an excellent opportunity to use this natural gum as a tablet binder, as has already been done in some studies [9,15].

Tablets prepared with okra gum have better physical properties compared to formulations prepared with pre-gelatinized starch, including lower friability and greater hardness. Similar results have also been reported in the literature [15], where okra gum in lower concentrations (1–5%) have produced tablets with acceptable properties of friability, hardness, and disintegration time, as compared with higher concentrations of PVP (22%) and cornstarch (12.5%). This might indicate a better binding strength of okra, as compared with starch in the same concentrations, as discussed before. The disintegration time of the tablet prepared with okra gum is relatively higher, which is obviously owing to its greater binding strength. Therefore, its concentration needs to be carefully adjusted when used as tablet excipient along with the addition of a disintegrant. The release of the drug from the tablets containing okra gum without a disintegrant (F1–F3) was slow, with a ~50% release in the first 30 min of dissolution, as compared with >80% release from the formulation (F7–F9) containing starch. In the okra formulation with a disintegrant (F4–F6), the release with still slow ~75% in 30 min, whereas such formulations of starch (F10–F12) have shown almost 100% drug release in the same time. This indicated the retardant nature of okra gum as already reported in literature [23].

## 5. Conclusions

It was concluded from this study that okra gum has a higher binding/adhesion strength as compared to pre-gelatinized starch. It employs that okra gum may be a better binder than pre-gelatinized starch and can be used as a binder in the future. This was also demonstrated by making tablets of naproxen sodium as a model drug.

## Figures and Tables

**Table 1:** Formulation of tablets prepared with okra gum and starch paste as a binder in different concentrations.

Formulation Name	Naproxen Sodium (mg)	Okra Gum (% *w*/*w*)	Starch Slurry (% *w*/*w*)	Disintegrant Starch (% *w*/*w*)	Magnesium Stearate (% *w*/*w*)	Talc (% *w*/*w*)	Lactose (mg)	Total Weight of Tablet (mg)
F1	275	1	-	-	1	0.5	168	450
F2	275	3	-	-	1	0.5	163	450
F3	275	5	-	-	1	0.5	157	450
F4	275	1	-	7	1	0.5	149	450
F5	275	3	-	7	1	0.5	143	450
F6	275	5	-	7	1	0.5	138	450
F7	275	-	1	-	1	0.5	168	450
F8	275	-	3	-	1	0.5	163	450
F9	275	-	5	-	1	0.5	157	450
F10	275	-	1	7	1	0.5	149	450
F11	275	-	3	7	1	0.5	143	450
F12	275	-	5	7	1	0.5	138	450

**Table 2:** Binding strength results of okra gum and pre-gelatinized starch as measured by double pulley assembly and universal testing machine.

Dispersion Sample Containing	Binding Strength (N)	
	Double Pulley Method	Universal Testing Method
Okra gum		
1%	1.44 ± 0.21	2.51 ± 0.18
3%	2.26 ± 1.52	3.63 ± 0.31
5%	2.42 ± 0.95	4.51 ± 0.49
Pre-gelatinized starch		
1%	1.00 ± 0.32	1.34 ± 0.45
3%	1.14 ± 0.25	1.48 ± 0.24
5%	1.73 ± 0.34	1.81 ± 0.53

**Table 3:** Bulk properties of granules prepared by using okra gum or starch as a binder.

Formulation	Bulk Density (g/cm^2^)	Tapped Density (g/cm^2^)	Hausner Ratio	Compressibility Index
F1	0.42	0.46	1.10	13
F2	0.43	0.48	1.12	10
F3	0.43	0.48	1.12	10
F4	0.43	0.48	1.12	10
F5	0.42	0.45	1.07	7
F6	0.43	0.45	1.05	4
F7	0.48	0.56	1.17	14
F8	0.45	0.53	1.18	15
F9	0.5	0.55	1.10	9
F10	0.5	0.56	1.12	5
F11	0.47	0.52	1.11	10
F12	0.48	0.53	1.10	9

**Table 4:** Values of friability, hardness, and disintegration time of formulations prepared using okra gum of pre-gelatinized starch.

Tablets Prepared with Okra Gum				Tablets Prepared with Pre-Gelatinized Starch			
Formulation Name	Friability (%)	Hardness (Kg/cm^2^)	Disintegration Time (min)	Formulation Name	Friability (%)	Hardness (Kg/cm^2^)	Disintegration Time (min)
**F1**	0.50	7.0 ± 0.5	8.10 ± 0.8	**F7**	0.62	6.5 ± 0.25	6.41 ± 0.1
**F2**	0.41	8.0 ± 0.5	11.30 ± 0.6	**F8**	0.51	7.0 ± 0.5	9.40 ± 1.0
**F3**	0.41	8.0 ± 0.6	15.00 ± 0.9	**F9**	0.54	8.0 ± 0.4	14.30 ± 1.0
**F4**	0.46	7.0 ± 1.0	7.51 ± 0.3	**F10**	0.69	7.0 ± 0.25	5.05 ± 0.2
**F5**	0.31	8.0 ± 1.0	11.04 ± 0.6	**F11**	0.62	7.5 ± 1.0	11.00 ± 0.5
**F6**	0.15	8.75 ± 0.3	9.18 ± 0.7	**F12**	0.62	7.75 ± 0.9	12.24 ± 0.7
